# Stochastic expansion of radionuclide inhalation dosimetry for consequence management application: uncertainty and sensitivity analysis in the ICRP 130 human respiratory tract model

**DOI:** 10.1088/1361-6498/ae81be

**Published:** 2026-07-14

**Authors:** Emmanuel Matey Mate-Kole, Sara C Howard, Ashley P Golden, David A Hooper, Brandon A Wilson, Shaheen Azim Dewji

**Affiliations:** 1 Nuclear and Radiological Engineering and Medical Physics Programs, George W. Woodruff School of Mechanical Engineering, Georgia Institute of Technology, Atlanta, GA, United States of America; 2 Epidemiology and Exposure Science, Oak Ridge Associated Universities, Oak Ridge, TN, United States of America; 3 Oak Ridge National Laboratory, 1 Bethel Valley Road, Oak Ridge, TN 37830, United States of America

**Keywords:** stochastic expansion, uncertainty propagation, sensitivity analysis, biokinetic models, HRTM, machine learning, internal dosimetry

## Abstract

Releases from nuclear or radiological security events can result in significant internal radiation contamination through inhalation of particulate contaminants. The reference human respiratory tract model (HRTM), developed by the International Commission on Radiological Protection (ICRP) and described in the ICRP Publication 66 with subsequent updates in the ICRP Publication 130, is used to evaluate the deposition and clearance behaviour of inhaled radionuclides. Biokinetic models are used to quantify the retention and excretion of internally deposited particulates, supporting the calculation of inhalation dose coefficients (DCs). The HRTM developed by the ICRP utilises deterministic quantities outlined in the ICRP Publication 66 and 130. The overarching goal of this study was to determine the variability from deterministic biokinetic/dosimetry models to represent the stochastic breadth of radionuclide metabolism in an exposed occupational population from realistic source terms, yielding an expanded compendium of inhalation DCs. The analysis was carried out in three phases: (1) Implementation and extension of the biokinetic and DC modelling framework based on the ICRP Publication 130 HRTM and associated element specific systemic biokinetic models; (2) Investigation of uncertain parameters in the HRTM; and (3) Stochastic analysis using Latin hypercube sampling, incorporating non-parametric (Kolmogorov–Smirnov statistics) test and quantile–quantile plot assessment to support parametric distribution selection, for characterisation of the committed effective DCs distributions. To determine the most impactful parameters among the uncertain parameters, a random forest regression model was employed for feature importance, coupled with SHapley Additive exPlanations for comprehensive machine learning interpretation of the features. This study presents a unique stochastic framework for modelling inhaled particulate metabolism, enhancing capabilities in radiation consequence management, medical countermeasure development, and radiation dose reconstruction for epidemiological investigations.

## 1. Introduction

The field of internal dosimetry addresses the assessment of radiation dose associated with the uptake, retention, and transport of radionuclides in tissues and organs within the body (Zanzonico 2000, Mate-Kole and Dewji 2024). Notable means of incorporation of radionuclides in the body include inhalation, ingestion, injection, and through the skin (Li 2018). Following entry via the respiratory or gastrointestinal pathways, radionuclides absorbed into the bloodstream may be transported to and redistributed among various organs and tissues, with subsequent removal occurring through urinary and faecal excretion, exhalation, and dermal pathways. Because direct measurement of radionuclide activity in organs is generally not feasible, internal dosimetry depends on biokinetic models, defined as mathematical frameworks that describe the temporal processes governing uptake, retention, redistribution, and clearance of radionuclides within biological systems (Bertelli *et al* 1997).

In biokinetic modelling, the transport of radionuclides or contaminants into, within, and out of the body is represented mathematically using systems of coupled ordinary differential equations (Li 2018). The organs and tissues included in these models act as radiation source regions, both physiologically and radiologically, as a result of ongoing uptake and retention of incorporated materials. Radioactive decay occurring within these source regions leads to irradiation of surrounding target tissues, resulting in energy deposition within the body. Within the framework established by the International Commission on Radiological Protection (ICRP) (ICRP 2007), radiation dose serves as a surrogate metric for assessing stochastic health risk. As a result, the mean absorbed dose to a target organ is determined by combining the specific absorbed fraction, defined as the fraction of energy emitted by a given radiation type in a source organ that is absorbed per unit mass of a target organ, with the radiation weighting factor appropriate to that radiation, yielding the equivalent dose (ICRP 2015, Li 2018). Equivalent doses are subsequently multiplied by tissue weighting factors to obtain the effective dose (ICRP 1991). As outlined in ICRP Publication 130 (ICRP 2015), dose coefficients for this application may be expressed either as dose per unit intake of a radionuclide or as dose per unit activity content, where the content function represents the time-dependent retention or excretion of the incorporated radionuclide.

For inhalation exposures, the ICRP has established a reference framework for respiratory tract deposition and biokinetics, referred to as the human respiratory tract model (HRTM), together with its associated dosimetric approaches. This model originated from the deterministic formulations presented in the ICRP Publication 66 (ICRP 1994a) and was subsequently updated in the ICRP Publication 130 (ICRP 2015). As a result, the HRTM is based on standardised anatomical, structural, and physiological parameters. According to the ICRP Publication 130 (ICRP 2015), these reference parameters are specified as fixed values for use in dose calculations, without explicitly accounting for uncertainty (Mate-Kole *et al* 2024). They are intended to represent a reference person: an idealised individual for whom equivalent organ and tissue doses are obtained by averaging values derived from idealised male and female anatomical and physiological models specified by the ICRP for radiological protection purposes (ICRP 2002, 2015). While the reference person framework provides a practical and consistent basis for prospective radiological protection of the general population, it is recognised that only a limited fraction of individuals within an exposed population will closely correspond to this idealised representation (NCRP 1998). The use of fixed dose quantities (such as dose coefficients) for public and occupational protection therefore does not adequately capture population variability, introducing uncertainty in the interpretation of calculated dose values when applied across diverse demographic groups. In Annexes D and E of the ICRP Publication 66 (ICRP 1994a), the influence of uncertainty and variability on parameters within the respiratory tract model were examined. Drawing on experimental evidence from both animal and human studies, these annexes explore approaches for defining model parameters so that they are consistent with measurable physiological and physicochemical variables. Examples of such measurable quantities include particle dissolution rate as a function of surface area, particle mass and diameter, material density, and dissolution rate constants. Parameterising dissolution behaviour in terms of observable variables enables more transparent sensitivity analyses of the respiratory tract model. In addition, deposition within human respiratory tract regions was further investigated by representing regional deposition as filtration efficiencies, rather than relying solely on interpolation of tabulated deposition fractions provided in Annexe F of the ICRP Publication 66 (ICRP 1994a).

Early investigations by Bolch *et al* (2001) performed a systematic evaluation of parameter uncertainties associated with the human respiratory tract deposition model described in the ICRP Publication 66. That study employed the Lung Dose Uncertainty Code (LUDUC), originally developed by Huston (1995), to quantify uncertainty in particle deposition. However, the LUDUC source code is no longer accessible, and its legacy computational architecture is incompatible with modern programming platforms. Building on this earlier work, Bolch *et al* (2003) examined uncertainty propagation within the ICRP Publication 66 respiratory tract model by introducing probabilistic representations for selected clearance parameters. Although this effort represented an important advancement in characterising uncertainty in internal dose assessment for inhalation scenarios, its scope was limited to particle mechanical clearance and to specific aerosol types, namely ^239^PuO_2_ and ^238^UO_2_/^238^U_3_O_8_. Subsequently, Huston *et al* (2003) expanded upon these analyses by performing a sensitivity study of the ICRP Publication 66 HRTM, incorporating the uncertain deposition and clearance parameters proposed in the earlier investigations. Nevertheless, their evaluation of equivalent dose per unit activity remained restricted to inhalation exposures involving monodisperse aerosol of ^239^PuO_2_. A recent study by Mate-Kole *et al* (2025) propagated uncertainty in committed effective dose coefficients (CEDCs) derived using the ICRP Publication 66 HRTM by examining the influence of selected respiratory tract uncertain input parameters for six radionuclides relevant to the Federal Radiological Monitoring and Assessment Center. The analysis considered adult inhalation exposures across specific lung absorption types such as fast (F), moderate (M), and slow (S), and spanned a range of particle size distributions, with activity median aerodynamic diameters (AMADs) between 0.003 *μ*m and 30 *μ*m. Although published earlier, Mate-Kole *et al* (2024) investigated uncertainty and sensitivity in the human respiratory tract using the updated ICRP Publication 130 HRTM in conjunction with a machine learning framework. In contrast to the study by Mate-Kole *et al* (2025), which employed the earlier ICRP Publication 66 model, the 2024 work (Mate-Kole *et al* 2024) leveraged the internal dosimetry toolkit [Radiological Exposure Dose Calculator (REDCAL)] and implemented the ICRP Publication 130 model but was limited in scope to inhalation exposure of ^131^I type F.

The objective of the present study is to extend the previously developed stochastic HRTM framework (Mate-Kole *et al* 2024) to incorporate multiple radionuclides, absorption types, and consequence management relevant source terms, resulting in a probabilistic compendium of inhalation dose coefficients. Building upon the earlier work, the stochastic analysis presented herein evaluates the variability inherent in conventional deterministic particle deposition and biokinetic models with associated dosimetric framework across multiple radionuclides following realistic nuclear security related intake scenarios. The analysis explicitly accounts for radionuclide inventory, particle size distribution, morphology, solubility, and clearance, and is implemented using the updated HRTM (the ICRP Publication 130 HRTM). Thus, this work provides an updated and expanded assessment relative to earlier investigations which were based on the ICRP Publication 66. The study is structured into three phases primarily, which are described below. In Phase 1, a computational framework is established for simulating respiratory tract deposition, systemic biokinetics, and the derivation of inhalation dose coefficients for occupational scenarios, with extension to public exposure conditions. This framework leverages the updated ICRP Publication 130 HRTM and integrates element-specific systemic biokinetic models within a Python-based implementation. In contrast to earlier work (Mate-Kole *et al* 2024), which focused on the development of a modular REDCAL framework applied to a limited radionuclide case study, the present implementation generalises the framework to support an expanded set of radiological and nuclear source terms, chemical forms, and exposure conditions. The computational module enables deterministic estimation of particle deposition, radionuclide retention, excretion, and dose coefficients. This expanded capability constitutes the deterministic core of REDCAL (REDCAL_det_), which serves as the foundation for subsequent uncertainty and sensitivity analyses for multiple radionuclides. Phase 2 examines uncertainty in model parameters relevant to the ICRP Publication 130 HRTM. This phase involved a comprehensive literature review to identify, categorise, and define probable bounds and statistical characteristics of probability distributions associated with the reference parameters implemented in Phase 1. Phase 3 applies stochastic analysis techniques to particle deposition and biokinetic modelling of nuclear security relevant source terms using the ICRP Publication 130 HRTM. In this phase, the deterministic computational framework established in Phase 1 is extended by incorporating probabilistic sampling of uncertain HRTM parameters through a Latin hypercube sampling (LHS) approach. This enables systematic evaluation of parameter sensitivity and uncertainty propagation within the dose assessment framework and constitutes the stochastic implementation of the REDCAL (REDCAL_stoch)_. Phase 3 further incorporates machine learning techniques for sensitivity analysis.

## 2. Methods

The method is organised into three primary phases aimed at establishing a mathematical and computational framework for stochastic biokinetic modelling of inhalation exposures arising from nuclear security related source terms. The framework accounts for radionuclide inventories, aerosol particle size distributions and morphology, solubility characteristics, and clearance behaviour, and is designed to support the derivation of statistically expanded inhalation dose coefficients. The study was conducted for occupational exposure and for an AMAD of 5 *μ*m.

### 2.1. Phase 1

Phase 1 focused on the implementation and extension of the REDCAL computational framework to support the estimation of inhalation dose coefficients for an expanded set of radiological and nuclear source terms. This phase encompassed the formulation and characterisation of source terms representative of nuclear security scenarios, followed by the calculation of corresponding dose coefficients. Central to this effort was the reconstruction and implementation of the updated ICRP Publication 130 HRTM together with the relevant systemic biokinetic models, implemented within a Python-based environment to enable comprehensive internal dose assessment. Table 1 provides the list of radionuclides considered and their corresponding absorption types. The radionuclides considered represent source terms associated with radiological dispersal devices (RDDs), nuclear power plants (NPPs), radioisotope thermoelectric generators (RTGs), and nuclear weapons (NWs), where NW debris include both state and hypothetical non-state weapons (sometimes commonly referred to as improvised nuclear devices, INDs).

**Table 1.** Selected radionuclides, absorption type and scenarios.

Radionuclides	Absorption type	Scenario	Justification and sources for absorption type selection
^60^Co	M	RDD	Type F is said to be unlikely in inhalation scenarios (ICRP 2016). Type M is default recommendation by ICRP in case the form is unknown (ICRP 2016).
^137^Cs	F	RDD/NW/NPP	Recommended type by the ICRP is F (caesium chloride, nitrate, sulphate) (ICRP 2017). Also, the National Research Council 2008 (NRC 2008)—Radiation source use and replacement recommended that the most commonly encountered form of caesium is caesium chloride.
^137^Cs	S	NW	Slow (S) absorption type is mostly recommended for nuclear weapons related (Hooper 2024). Thus, for NW in particular, if there is a surface burst, the isotopes will only make up about 1 × 10^−8^ of the debris. The particles will have the characteristics of the carrier material, which is often high in aluminosilicates and will vitrify.
^241^Am	M	RDD	Americium oxide (type M)—Selected based on common form of sources (NRC 2008) and in DTIC Technical Report (Stricklin *et al* 2014). ICRP 141 (ICRP 2019) default recommendation is type M when unknown or with limited information.
^241^Am	S	NW	Type S is Americium with plutonium oxide (ICRP 2019). Slow (S) absorption type is mostly recommended for nuclear weapons related (Hooper 2024). Thus, for NW in particular, if there is a surface burst, the isotopes will only make up about 1 × 10^−8^ of the debris. The particles will have the characteristics of the carrier material, which is often high in aluminosilicates and will vitrify.
^90^Sr	M	RDD/NW/NPP	Type M is recommended for irradiated fuel fragments and any unknown chemical form or forms with limited information (ICRP 2016).
^90^Sr	S	RDD/NW	Type S—Strontium titanate was shown to be retained tenaciously in the human lungs (ICRP 2016). Also referenced as common form recommended by the National Research Council report (NRC 2008). Slow (S) absorption type is mostly recommended for nuclear weapons related (Hooper 2024). Thus, for NW in particular, if there is a surface burst, the isotopes will only make up about 1 × 10^−8^ of the debris. The particles will have the characteristics of the carrier material, which is often high in aluminosilicates and will vitrify.
^89^Sr	M	NW/NPP	Type M is recommended for irradiated fuel fragments and any unknown chemical form or forms with limited information (ICRP 2016).
^89^Sr	S	NW	Type S-Strontium titanate was shown to be retained tenaciously in the human lungs (ICRP 2016). Also referenced as common form recommended by the National Research Council report (NRC 2008). Slow (S) absorption type is mostly recommended for nuclear weapons related (Hooper 2024). Thus, for NW in particular, if there is a surface burst, the isotopes will only make up about 1 × 10^−8^ of the debris. The particles will have the characteristics of the carrier material, which is often high in aluminosilicates and will vitrify.
^90^Y	M	NPP	National Research Council recommended oxide form as most commonly encountered (NRC 2008). ICRP134 (ICRP 2016) recommended oxide form of yttrium as type M.
^90^Y	S	RDD/NW	Yttrium in a form of fused aluminosilicate particles—type S (ICRP 2016). It is also been used in inhalation studies (Lay *et al* 1975). Slow (S) absorption type is mostly recommended for nuclear weapons related (Hooper 2024). Thus, for NW in particular, if there is a surface burst, the isotopes will only make up about 1 × 10^−8^ of the debris. The particles will have the characteristics of the carrier material, which is often high in aluminosilicates and will vitrify.
^91^Y	M	NPP	National Research Council recommended oxide form as most commonly encountered (NRC 2008). ICRP134 (ICRP 2016) recommended oxide form of yttrium as type M.
^91^Y	S	RDD/NW	Yttrium in a form of fused aluminosilicate particles—type S (ICRP 2016). It is also been used in inhalation studies (Lay *et al* 1975). Slow (S) absorption type is mostly recommended for nuclear weapons related (Hooper 2024). Thus, for NW in particular, if there is a surface burst, the isotopes will only make up about 1 × 10^−8^ of the debris. The particles will have the characteristics of the carrier material, which is often high in aluminosilicates and will vitrify.
^131^I	S	NW	Slow (S) absorption type is mostly recommended for nuclear weapons related (Hooper 2024). Thus, for NW in particular, if there is a surface burst, the isotopes will only make up about 1 × 10^−8^ of the debris. The particles will have the characteristics of the carrier material, which is often high in aluminosilicates and will vitrify.
^133^I	F	NW/NPP	Recommended type by the ICRP is F as default for sodium iodide; caesium chloride vector, silver iodide and also for any form that is unknown or known but with no information regarding the absorption of that form from the respiratory tract (ICRP 2017).
^133^I	S	NW	Slow (S) absorption type is mostly recommended for nuclear weapons related (Hooper 2024). Thus, for NW in particular, if there is a surface burst, the isotopes will only make up about 1 × 10^−8^ of the debris. The particles will have the characteristics of the carrier material, which is often high in aluminosilicates and will vitrify.
^134^Cs	F	NW/NPP	Recommended type by the ICRP is F (caesium chloride, nitrate, sulphate) (ICRP 2017). Also, the National Research Council 2008—Radiation source use and replacement recommended that the most commonly encountered form of caesium is caesium chloride (NRC 2008).
^134^Cs	S	NW	Slow (S) absorption type is mostly recommended for nuclear weapons related (Hooper 2024). Thus, for NW in particular, if there is a surface burst, the isotopes will only make up about 1 × 10^−8^ of the debris. The particles will have the characteristics of the carrier material, which is often high in aluminosilicates and will vitrify.
^144^Ce	M	NW/NPP	Default type M is recommended for use in the absence of specific information on which the exposure material can be assigned to an absorption type; for example, if the form is unknown, or if the form is known but there is no information available on the absorption of that form from the respiratory tract (ICRP 2019).
^144^Ce	S	NW	Slow (S) absorption type is mostly recommended for nuclear weapons related (Hooper 2024). Thus, for NW in particular, if there is a surface burst, the isotopes will only make up about 1 × 10^−8^ of the debris. The particles will have the characteristics of the carrier material, which is often high in aluminosilicates and will vitrify.
^95^Nb	M	NPP/NW/RDD	Default type M is recommended for use in the absence of specific information on which the exposure material can be assigned to an absorption type; for example, if the form is unknown, or if the form is known but there is no information available on the absorption of that form from the respiratory tract. Irradiated fuel, nuclear weapons fallout and oxalate are consistent with type M (ICRP 2016).
^95^Nb	S	NW	Nuclear weapons fallout of Nb in particulate form is consistent with type M or S (ICRP 2016). Slow (S) absorption type is mostly recommended for nuclear weapons related (Hooper 2024). Thus, for NW in particular, if there is a surface burst, the isotopes will only make up about 1 × 10^−8^ of the debris. The particles will have the characteristics of the carrier material, which is often high in aluminosilicates and will vitrify.
^144^Nd	M	NPP/RDD	Default type M is recommended for use (absorption parameter values for inhaled and ingested lanthanides) in the absence of specific information on which the exposure material can be assigned to an absorption type; for example, if the form is unknown, or if the form is known but there is no information available on the absorption of that form from the respiratory tract (ICRP 2019).
^238^Pu	M	NPP/RDD/RTG	Default type M is recommended for use in the absence of specific information on which the exposure material can be assigned to an absorption type; for example, if the form is unknown, or if the form is known but there is no information available on the absorption of that form from the respiratory tract (ICRP 2019).
^238^Pu	S	NW/RDD	Slow (S) absorption type is mostly recommended for nuclear weapons related (Hooper 2024). Thus, for NW in particular, if there is a surface burst, the isotopes will only make up about 1 × 10^−8^ of the debris. The particles will have the characteristics of the carrier material, which is often high in aluminosilicates and will vitrify.
^239^Pu	M	RDD/RTG	Default type M is recommended for use in the absence of specific information on which the exposure material can be assigned to an absorption type; for example, if the form is unknown, or if the form is known but there is no information available on the absorption of that form from the respiratory tract (ICRP 2019).
^239^Pu	S	NW/RDD	Slow (S) absorption type is mostly recommended for nuclear weapons related (Hooper 2024). Thus, for NW in particular, if there is a surface burst, the isotopes will only make up about 1 × 10^−8^ of the debris. The particles will have the characteristics of the carrier material, which is often high in aluminosilicates and will vitrify.
^235^U	Intermediate type M/S: uraniumoctoxide U_3_O_8_	NPP/NW/RDD	Default type M is recommended for use in the absence of specific information on which the exposure material can be assigned to an absorption type; for example, if the form is unknown, or if the form is known but there is no information available on the absorption of that form from the respiratory tract (ICRP 2017). Note: Irradiated fuels are also consistent with type M as stated in ICRP 137 for uranium. But U_3_O_8_ is assigned intermediate type M/S.

Further, it is important to note that the deterministic inhalation dose coefficients were derived in accordance with recommendations issued by the ICRP (ICRP 2007, 2015). The computational module is intended to implement methodologies of occupational intake of radionuclides and intake of radionuclides by members of the public. However, this study reports analysis conducted for occupational intake, where 50 years was employed as the committed period. The resulting biokinetic formulations give rise to complex mathematical systems that can be solved using either numerical or analytical approaches. Detailed mathematical evaluation and benchmark of such biokinetic models have been presented elsewhere (Mate-Kole *et al* 2023, Mate-Kole and Dewji 2024) and the suitable solvers were adopted in the framework of this study. Following the calculation of radionuclide retention and excretion for each organ, designated as source organs within the biokinetic models, the corresponding time-integrated activity is determined over a 50 year commitment period for occupational intakes. The radiation dose to a given target organ is then obtained by combining this time-integrated activity with the appropriate time-independent dosimetric factors, yielding the committed equivalent dose coefficient. The committed equivalent dose coefficients are subsequently multiplied by tissue weighting factors for the respective target organs to derive the CEDCs (ICRP 2007).

Additionally, the dose coefficients for internally incorporated radionuclides reflect not only the radiation emitted by the parent nuclide but also the contributions from radioactive progeny produced *in vivo* following intake. As a result, the precision of these dose coefficients is strongly influenced by the assumptions embedded within the biokinetic models regarding the transport, retention, and distribution of the decay products, where applicable. For inhalation scenarios, radionuclides that are generated prior to intake and inhaled concurrently with the parent nuclide are treated as independent intakes, with each progeny radionuclide assumed to follow the element-specific biokinetic model. In this study, radioactive progeny produced within the respiratory tract are modelled in accordance with the recommendations of ICRP Publication 130 (ICRP 2015), as described below:

a) The rate at which a particle dissociates is determined by the particle matrix, therefore shared kinetics is assumed for dissolution parameters except noble gases like radon.
b) Independent kinetics for bound parameters because the behaviour of dissociated material would depend on its elemental form.
c) The fractional absorption in the alimentary tract for relatively soluble forms of the element (*f*_A_) is taken to be that of the progeny radionuclide.

In the ICRP Publication 30 and 68 (ICRP 1979, 1994b), decay products produced within systemic compartments were, by default, assumed to share the same biokinetic behaviour as the parent radionuclide. This approach is commonly referred as shared kinetics. The ICRP Publication 68 (ICRP 1994b), however, also proposed an alternative treatment for selected parent radionuclides, whereby radioactive progeny were assumed to follow element-specific, independent biokinetics. Under this framework, the systemic transport and retention of *in-vivo* generated progeny are determined by their own biological and systemic characteristics rather than those of the parent nuclide. The present study adopted the independent-kinetics assumption for decay chain members, where applicable.

**Figure 1.**
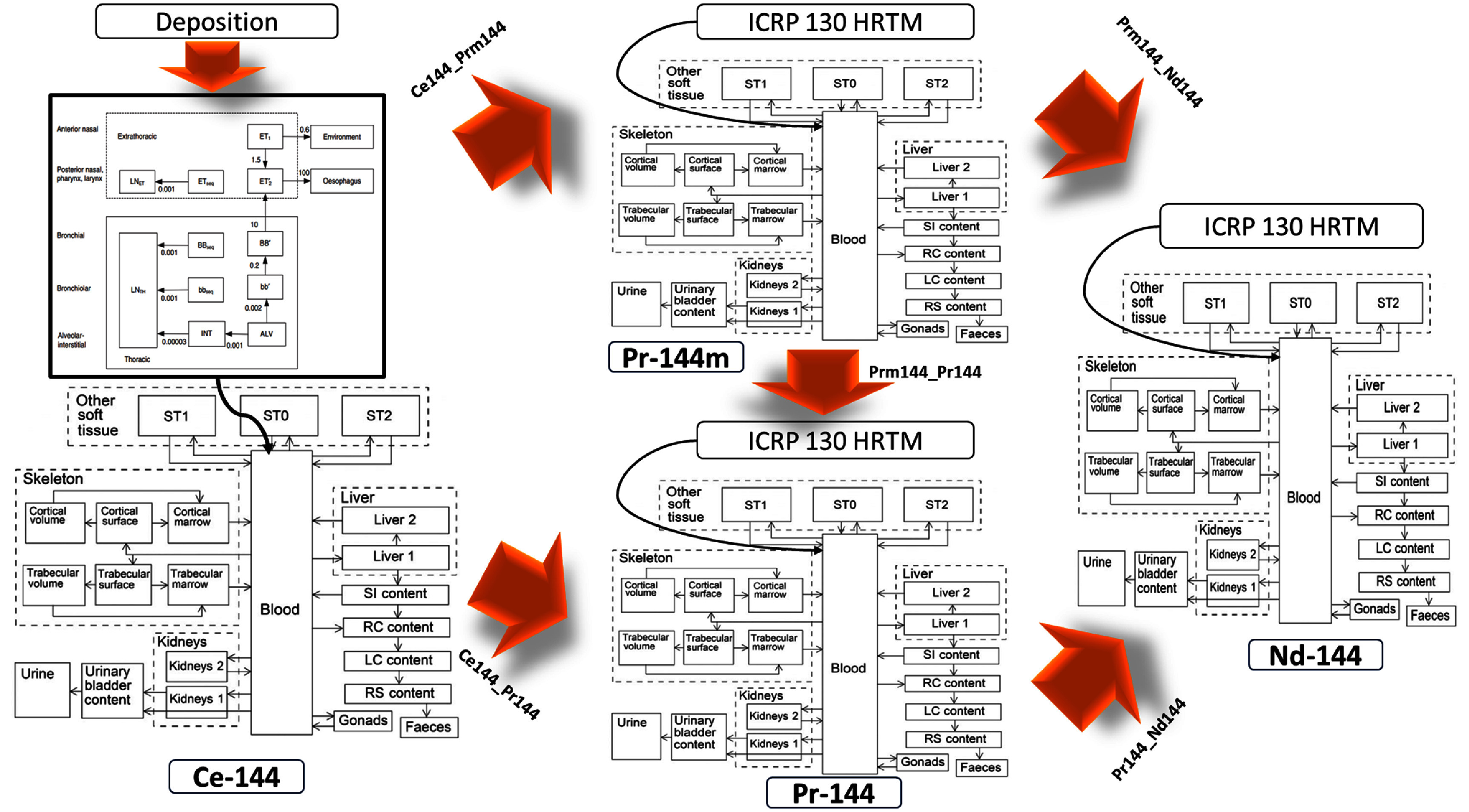
Biokinetic modelling with decay chain for inhaled ^144^Ce (ICRP 2015, 2019). HRTM: human respiratory tract model; HATM: human alimentary tract model; ST: soft tissue; SI: small intestine; RC: right colon; LC: left colon; RS: rectosigmoid colon. The figure for the systemic model of Cerium was reproduced with permission from the ICRP Publication 141, figure 2.7, and the figure for the HRTM was reproduced with permission from the ICRP Publication 130, figure 3.4. Reproduced with permission from ICRP (2019). Reproduced with permission from ICRP (2015).

The application of independent-kinetics assumptions to radionuclide decay chains is relatively straightforward when biokinetic models for all chain members share a common structural framework, including consistent compartment definitions. Under these conditions, each progeny nuclide is represented using its own element-specific biokinetic model. This situation commonly arises for decay chains involving lanthanide radioisotopes whose progeny are also lanthanides, with notable exceptions in certain lanthanum or cerium decay series where caesium and barium isotopes are present, as described in ICRP Publication 141. Figure 1 illustrates the radionuclide decay chain biokinetic model for inhaled ^144^Ce, in which the parent nuclide and its progeny share the same systemic biokinetic structural framework.

It is worth noting that implementing independent kinetics for members of a radionuclide decay chain introduces additional modelling complexity, particularly when the biokinetic models of the parent and progeny nuclides are structurally different. In some cases, decay products may be generated in source regions that are not explicitly represented in the progeny’s characteristic biokinetic model. When this occurs, it is necessary to define both the transfer rate from the region of origin and the destination compartment within the progeny model prior to solving the system. Furthermore, parent and daughter nuclides may differ in their compartmental representations. For example, a parent radionuclide biokinetic model may include a single kidney compartment, whereas the corresponding progeny model may subdivide the kidney into multiple compartments (e.g. ‘Kidney 1’ and ‘Kidney 2’). Such structural mismatches require additional remodelling to ensure precise formulation and solution of the biokinetic equations. These associated complexities were explicitly addressed in the present study for the applicable radionuclides.

### 2.2. Phase 2

The second phase of this work adopted the framework previously detailed by Mate-Kole *et al* (2024) for the investigation of uncertain parameters in the HRTM with respect to the ICRP Publication 130 HRTM. Briefly, uncertainty and variability in respiratory tract deposition, sequestration, breathing condition, and mechanical clearance processes were represented using probability distributions assigned to selected HRTM parameters. The uncertain parameters, associated probability distributions, and statistical descriptors used in the present study are summarised in table 2 and are identical to those reported by Mate-Kole *et al* (2024).

**Table 2.** Uncertain parameters incorporated for the stochastic analysis (Mate-Kole *et al* 2024).

Input	Description	Unit	Distribution	Mean/GM	SD/GSD
*Fn*	Fraction of air inhaled (Puncher 2014)	Unitless	Triangular	Mode = 1	Min: 0.4Max: 1
*Cae_ET1*	Random variable for aerodynamic deposition efficiency in *ET1* (Bolch *et al* 2001)	Unitless	Lognormal	1.00	1.82
*Cae_ET2*	Random variable for aerodynamic deposition efficiency in *ET2* (Bolch *et al* 2001)	Unitless	Lognormal	1.00	1.82
*Cae_BB*	Random variable for aerodynamic deposition efficiency in *BB* (Bolch *et al* 2001)	Unitless	Lognormal	1.00	1.58
*Cae_bb*	Random variable for aerodynamic deposition efficiency in *bb* (Bolch *et al* 2001)	Unitless	Lognormal	1.00	1.58
*Cae_AI*	Random variable for aerodynamic deposition efficiency in *AI* (Bolch *et al* 2001)	Unitless	Lognormal	1.00	1.3
*Cth_ET1*	Random variable for thermodynamic deposition efficiency in *ET1* (Bolch *et al* 2001)	Unitless	Lognormal	1.00	1.18
*Cth_ET2*	Random variable for thermodynamic deposition efficiency in *ET2* (Bolch *et al* 2001)	Unitless	Lognormal	1.00	1.18
*Cth_BB*	Random variable for thermodynamic deposition efficiency in *BB* (Bolch *et al* 2001)	Unitless	Lognormal	1.00	1.23
*Cth_bb*	Random variable for thermodynamic deposition efficiency in *bb* (Bolch *et al* 2001)	Unitless	Lognormal	1.00	1.23
*Cth_AI*	Random variable for thermodynamic deposition efficiency in *AI* (Bolch *et al* 2001)	Unitless	Lognormal	1.00	1.23
*fd_ETseq*	Fraction of deposit in *ETseq* compartment (Bolch *et al* 2003)	Unitless	Lognormal	0.002	1.73
*fd_BBseq*	Fraction of deposit in *BBseq* compartment (Bolch *et al* 2003)	Unitless	Lognormal	0.002	1.73
*fd_bbseq*	Fraction of deposit in *bbseq* compartment (Bolch *et al* 2003)	Unitless	Lognormal	0.002	1.73
*L_ALV, INT*	Fractional clearance rate constant for mechanical clearance from *ALV* to *INT* (Puncher and Burt 2013)	d^−1^	Lognormal	Median = 0.001	4.5
*L_ALV,bb*	Fractional clearance rate constant for mechanical clearance from *ALV* to *bb′* (Puncher and Burt 2013)	d^−1^	Lognormal	Median = 0.0013	3.2
*L_INT,LNTH*	Fractional clearance rate constant for mechanical clearance from *INT* to *LNTH* (Puncher and Burt 2013)	d^−1^	Lognormal	Median = 0.00003	3
*K_factor*	Randomly sampled factor (Puncher and Burt 2013) from the given distribution to scale the following rates (*ET1 ➔ ET2; ET1 ➔ Env; ETseq ➔ LNET; ET2 ➔ Oeso; BBseq ➔ LNTH; BB ➔ ET2; bbseq ➔ LNTH; bb ➔ BB*)	Unitless	Lognormal	Median of unity: 1	1.73

**Notation:** *Oeso*—Oesophagus, *Env*—Environment. *ET1*: anterior nose, *ETseq*: long-term retention within airway tissues of a small portion of particles deposited in the nasal passages, *ET2*: Extrathroacic posterior nasal region, *BB*: Bronchial region, *bb*: Bronchiolar region, *BBseq*: long-term retention in airway walls in *BB* region, *bbseq*: long-term retention in airway walls in bb region, *LNET*: lymphatics and lymph nodes that drain the extrathoracic (*ET*), *LNTH*: lymphatics and lymph nodes in the thoracic region, *ALV*: retention of particles deposited in the alveoli, *INT*: very long-term retention of the particles deposited in the alveoli that penetrate to the interstitium, the ‘′’ indicates short term retention in a compartment. The implemented LHS algorithm uses GM and GSD to define lognormal distributions; therefore, all tabulated lognormal distribution bounds were expressed in terms of these parameters.

The probability distributions and parameter bounds assigned to uncertain parameters of the HRTM were selected based on prior published studies and available experimental evidence describing the variability in respiratory tract deposition and clearance processes. The selected bounds were intended to represent population level variability within occupationally exposed subjects.

The uncertainty propagation conducted in this study is limited to variability and uncertainty associated with the HRTM, specifically respiratory tract deposition and clearance parameters. Systemic biokinetic transfer coefficients, gastrointestinal absorption fractions, and dosimetric S coefficients were kept fixed at ICRP reference values in the present framework.

### 2.3. Phase 3

**Figure 2.**
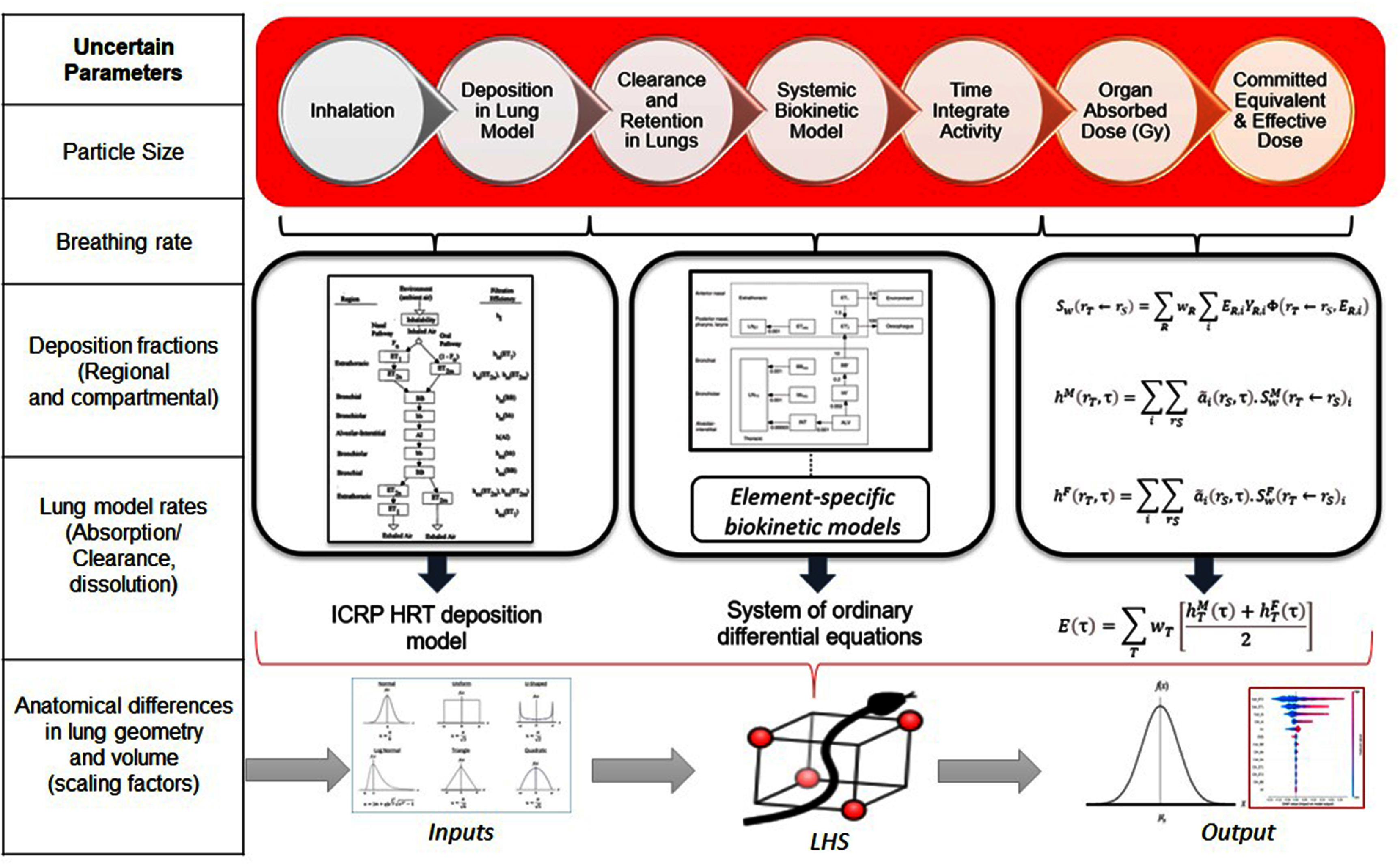
Inhalation dose coefficient computational framework incorporating uncertain parameters. The figure for the filtration mechanism illustrated in the particle deposition block was reproduced with permission from the ICRP Publication 66, figure 8, and the HRTM in the middle block was reproduced with permission from the ICRP Publication 130, figure 3.4. Reproduced with permission from ICRP (2015). Reproduced with permission from ICRP (1994a).

Phase 3 of the study focused on the application of stochastic methods to the particle deposition and biokinetic models developed for nuclear security relevant source term intakes, with the objective of deriving expanded inhalation dose coefficients for multiple radionuclides. An overview of the inhalation dose coefficient calculation module, including the incorporation of uncertain model parameters, is presented in figure 2.

The uncertainty propagation methodology employed in this study leveraged a sampling approach (LHS) previously described in a prior study (Mate-Kole *et al* 2024). Compared with conventional Monte Carlo methods, LHS typically achieves comparable statistical accuracy with a substantially smaller number of samples (McKay and Beckman 2000). A LHS size of 10 000 realisations was selected to provide sufficient resolution and statistical power, taking advantage of the more rapid reduction in sampling error afforded by LHS quadratically faster relative to conventional Monte Carlo methods for an equivalent number of samples. In a few select cases, the sample size was reduced to 5000 realisations where a smaller ensemble was deemed appropriate to avoid excessive statistical power. The uncertain parameters identified in Phase 2, together with their assigned probability distributions, were incorporated within a Python-based LHS framework and randomly sampled using pyDOE2 (Sjögren and Svensson 2018). For each radionuclide considered in Phase 1, inhalation CEDCs were subsequently evaluated across the ensemble of sampled parameter vectors.

It is important to note that no explicit sampling physiological correlation was imposed across parameters because the LHS framework assumed independent sampling. Therefore, some sampled combinations may represent less likely physiological combinations, even though individual parameter values were constrained within their prescribed literature supported ranges. These combinations were retained because the analysis was intended to characterise population level HRTM-driven uncertainty rather than individual-specific physiology.

To describe the distribution of the response variable, the CEDC, two complementary steps were undertaken: first, descriptive summary statistics were computed, and second, the resulting data were fitted to a set of candidate probability distributions. Statistical summary including the minimum, maximum, mean, standard deviation, and selected percentiles (25th, 50th, and 75th) were evaluated for each radionuclide and source term scenario. The estimated percentiles were subsequently compared with the corresponding reference ICRP dose coefficient to determine the location of the deterministic value within the simulated distribution. Uncertainty was quantified using the lower (2.5th percentile, ${Q_{\mathrm{L}}}$) and upper (97.5th percentile, ${Q_{\mathrm{U}}}$) bounds, defining an interval that encompasses approximately 95% of the distribution and is expected to contain the true central value. A central estimate, $C$, was then used to compute the uncertainty factor (UF), defined as the larger of the ratios $C/{Q_{\mathrm{L}}}$ and ${Q_{\mathrm{U}}}/C$ (Leggett 2001).

In addition, the Kolmogorov–Smirnov (K–S) test was applied to identify the most appropriate probability distribution for each inhalation scenario without assuming an underlying distribution for the response variable. The K–S test is a nonparametric goodness-of-fit method that evaluates whether a sample is consistent with a specified distribution by comparing their cumulative distribution functions (Aslam 2019). The test is formulated under the null hypothesis that the empirical sample and the reference distribution originate from the same distribution, using a two-sided criterion. This hypothesis is rejected when sufficient evidence indicates a statistically significant difference between the two distributions. In this study, the K–S statistic was computed for each candidate distribution using the *kstest* implementation in the SciPy library (Virtanen *et al* 2020). Specifically, the statistic corresponds to the maximum absolute difference, ${D_{{\mathrm{max}}}} = {\mathrm{max}}\mid {\mathrm{eCDF}} - {\mathrm{tCDF}}\mid $, between the empirical cumulative distribution function of the CEDCs and the corresponding theoretical cumulative distribution function. The distribution yielding the smallest value of ${D_{{\mathrm{max}}}}$ was therefore identified as the most suitable representation of the data. Associated p-values were obtained using the default one-sample, two-sided formulation, representing the probability of observing a test statistic at least as extreme as the computed value under the assumption that the result data follow the specified distribution (Moore *et al* 2009). A significance level of 0.05 was adopted, with p-values below this threshold interpreted as grounds for rejecting the null hypothesis. Inferred distributions of the CEDCs were further evaluated using quantile–quantile (Q–Q) plots as a complementary diagnostic approach. These plots compare empirical quantiles of the simulated data with theoretical quantiles from the candidate distribution. Agreement with the assumed distribution is indicated by points clustering along the 45° reference line, while departures from this line reflect deviations between the observed data and the theoretical model. The fitted response distributions presented in this study are intended primarily as descriptive statistical representations of the simulated dose coefficient outputs based on the case study and the uncertain parameters incorporated. The primary purpose is to characterise important features of the stochastic responses associated with uncertainty propagation. For practical applications, simpler distributions may suffice. Nevertheless, heavier-tailed fitted distributions remain valuable for highlighting upper-bound behaviour and low-probability high-consequence outcomes that may be particularly relevant for consequence management, emergency response planning, and retrospective dose reconstruction applications.

Moreover, to identify and rank the most influential model parameters, a random forest (RF) machine learning regression framework coupled with SHapley Additive exPlanations (SHAPs) analysis was employed to enhance interpretability and transparency of the sensitivity assessment. This approach was adopted from Mate-Kole *et al* (2024), where RF-SHAP analysis was introduced as an alternative to conventional sensitivity measures, such as standardised rank regression coefficients (Iman *et al* 1985, Huston 1995, Bolch *et al* 2001), for capturing nonlinear relationships and higher-order interactions among uncertain model parameters.

For each radionuclide and source term scenario considered in this study, the CEDC served as the target response variable. Prior to model training, CEDC values were normalised using min–max scaling to the interval [0,1]. The dataset was partitioned into training and testing subsets using a 75:25 split, and the optimal number of trees was determined through grid-search optimisation. The RF regression model was implemented using the *RandomForestRegressor* class in scikit-learn (Pedregosa *et al* 2011), and model performance was evaluated using the root mean squared error (RMSE) and mean absolute error (MAE). Following model training, SHAP analysis was performed to quantify parameter importance and support interpretation of both global sensitivity patterns and parameter specific contributions to variability in the resulting dose coefficient distributions.

## 3. Results

The result section summarises the uncertainty and sensitivity analysis performed for the expanded biokinetic and dosimetric framework, including the HRTM, used to derive inhalation dose coefficient distributions. The evaluation encompassed uncertainty propagation, statistical characterisation of the CEDC, and identification of influential model parameters. For illustrative purposes, a detailed presentation of results is provided for ^238^Pu (type M), while a comprehensive summary of findings for all radionuclides considered in this study is included as accompanying compendium of results.

Table 3 summarises the statistical descriptors for the ^238^Pu type M inhalation scenario at an AMAD of 5 *µ*m based on 10 000 realisations. Uncertainty in the CEDC is quantified using the 2.5th (*Q*_L_) and 97.5th (*Q*_U_) percentiles, which define the interval to contain approximately 95% of the distribution. This interval is expressed in terms of the corresponding UF. As shown in table 4, the ^238^Pu type M inhalation case yields a UF of 6.58, indicating that the central estimate is bounded within this range with approximately 95% confidence.

**Table 3.** Committed effective dose coefficient (CEDC) (*Sv*/*Bq*) response for inhaled ^238^Pu type M summary statistics.

Statistics	CEDC response
Mean	3.16 × 10^−05^
Std	1.96 × 10^−05^
Min	1.48 × 10^−06^
2.50th percentile	6.06 × 10^−06^
25th percentile	1.71 × 10^−05^
50th percentile	2.76 × 10^−05^
75th percentile	4.19 × 10^−05^
97.50th percentile	7.89 × 10^−05^
Max	2.07 × 10^−04^

Std: standard deviation; min: minimum; max: maximum.

**Table 4.** Inhalation committed effective dose coefficient (*Sv*/*Bq*) uncertainty bound for ^238^Pu type M for an AMAD of 5 *µ*m.

Quantity	Value
ICRP	1.20 × 10^−05^
*Q*_L_	6.06 × 10^−06^
*Q*_U_	7.89 × 10^−05^
*Q*_U_/ICRP	6.58
ICRP/*Q*_L_	1.98

**Figure 3.**
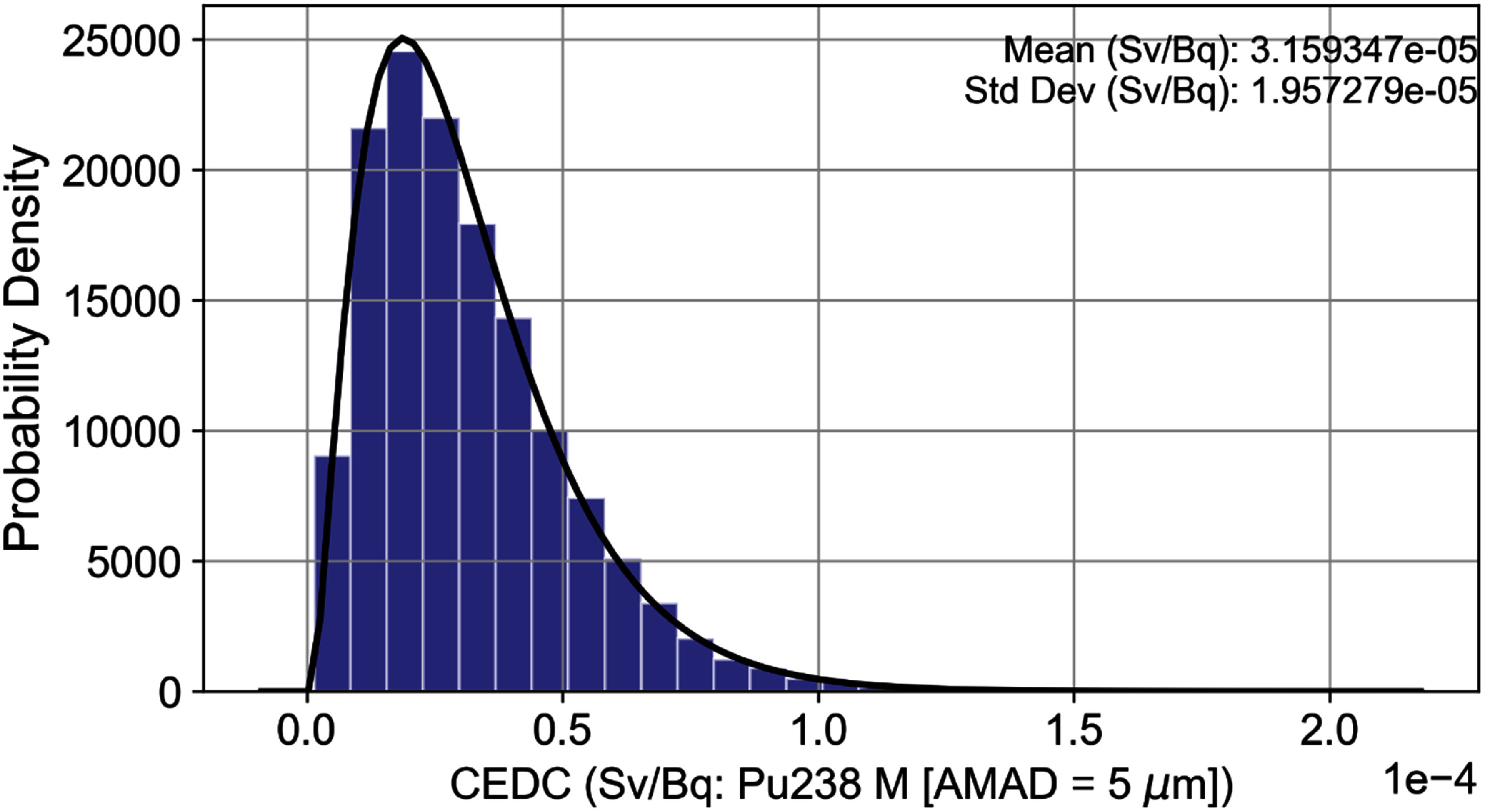
Best fit probability distribution (gamma) of the committed effective dose coefficient (CEDC) for inhaled ^238^Pu type M. Std Dev indicates the standard deviation of the CEDC samples.

**Figure 4.**
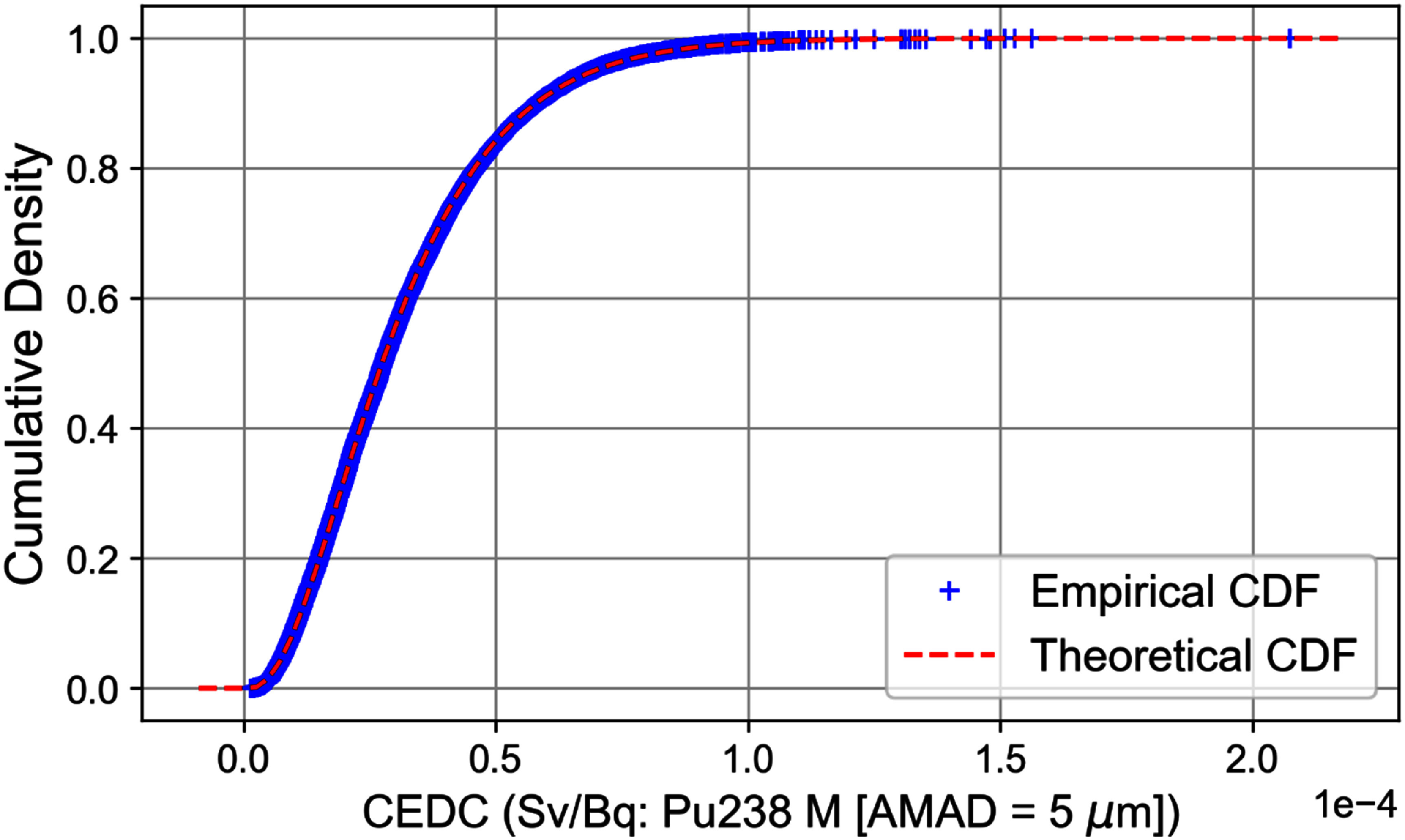
Cumulative density function plot for inhaled ^238^Pu type M comparing the underlying distribution of the committed effective dose coefficient (CEDC) and the predicted best fit distribution (gamma).

Figure 3 shows the probability density function corresponding to the best-fit distribution for the CEDC, identified as the gamma distribution using the nonparametric K–S test implemented in Python. Figure 4 presents the associated cumulative distribution comparison, juxtaposing the empirical CEDC data with the fit theoretical distribution across all 10 000 realisations.

**Figure 5.**
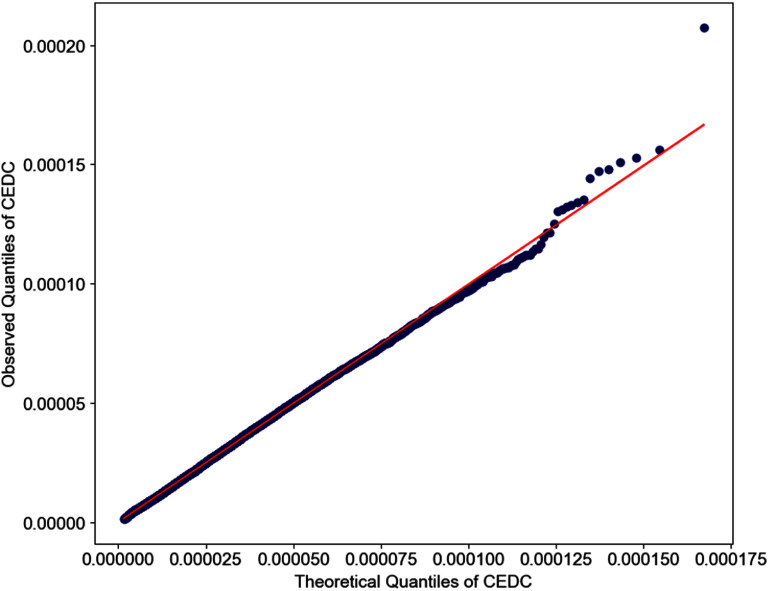
Q–Q plot of the best fit distribution (gamma) comparing the observed committed effective dose coefficient (CEDC) for ^238^Pu type M to the theoretical distribution.

The fitted distribution was additionally examined using Q–Q plot (figure 5) to evaluate the quality of the distributional fit.

**Figure 6.**
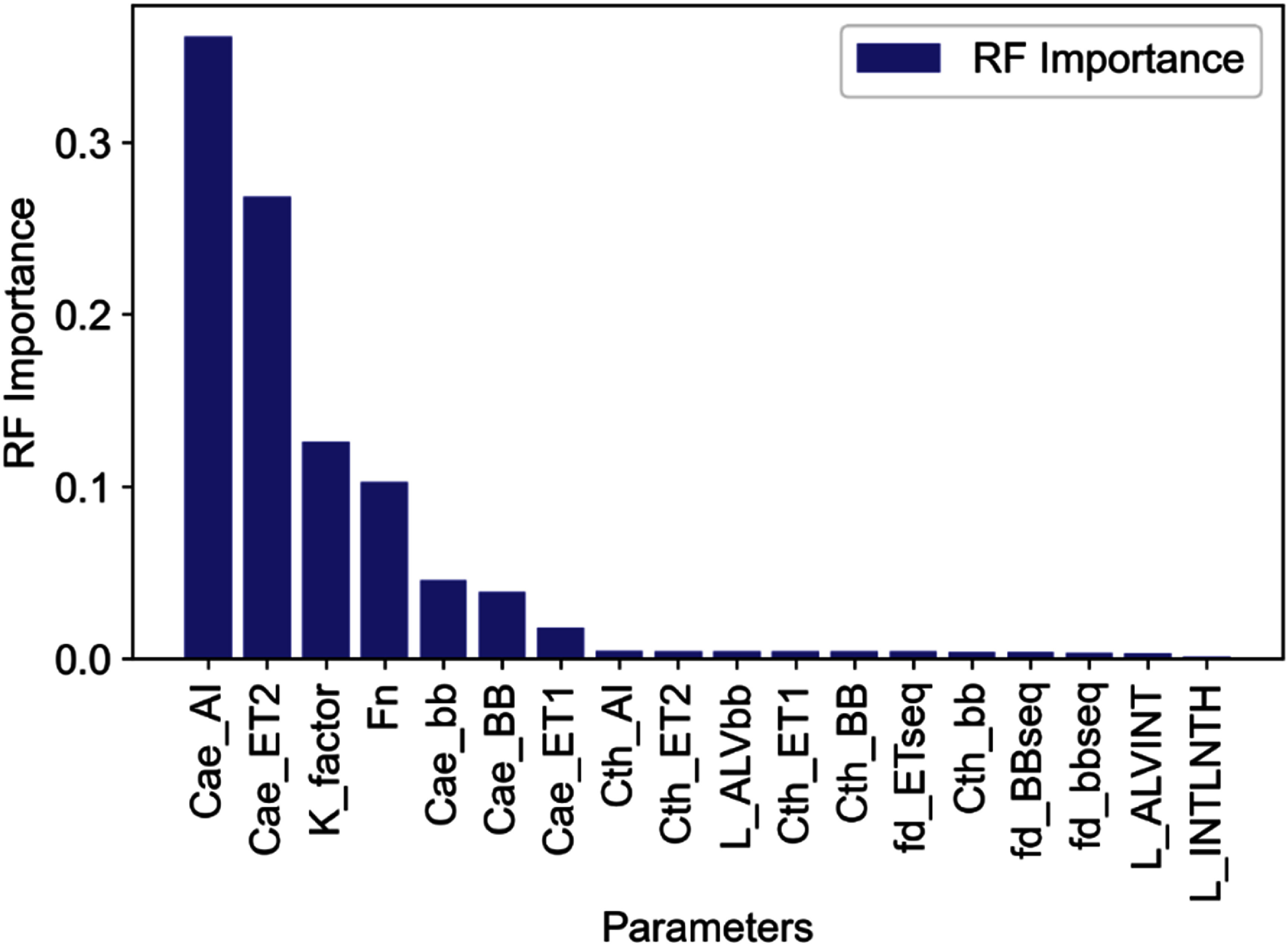
Random forest parameter importance ranking of uncertain parameters with the committed effective dose coefficient as the target response for ^238^Pu type M.

**Figure 7.**
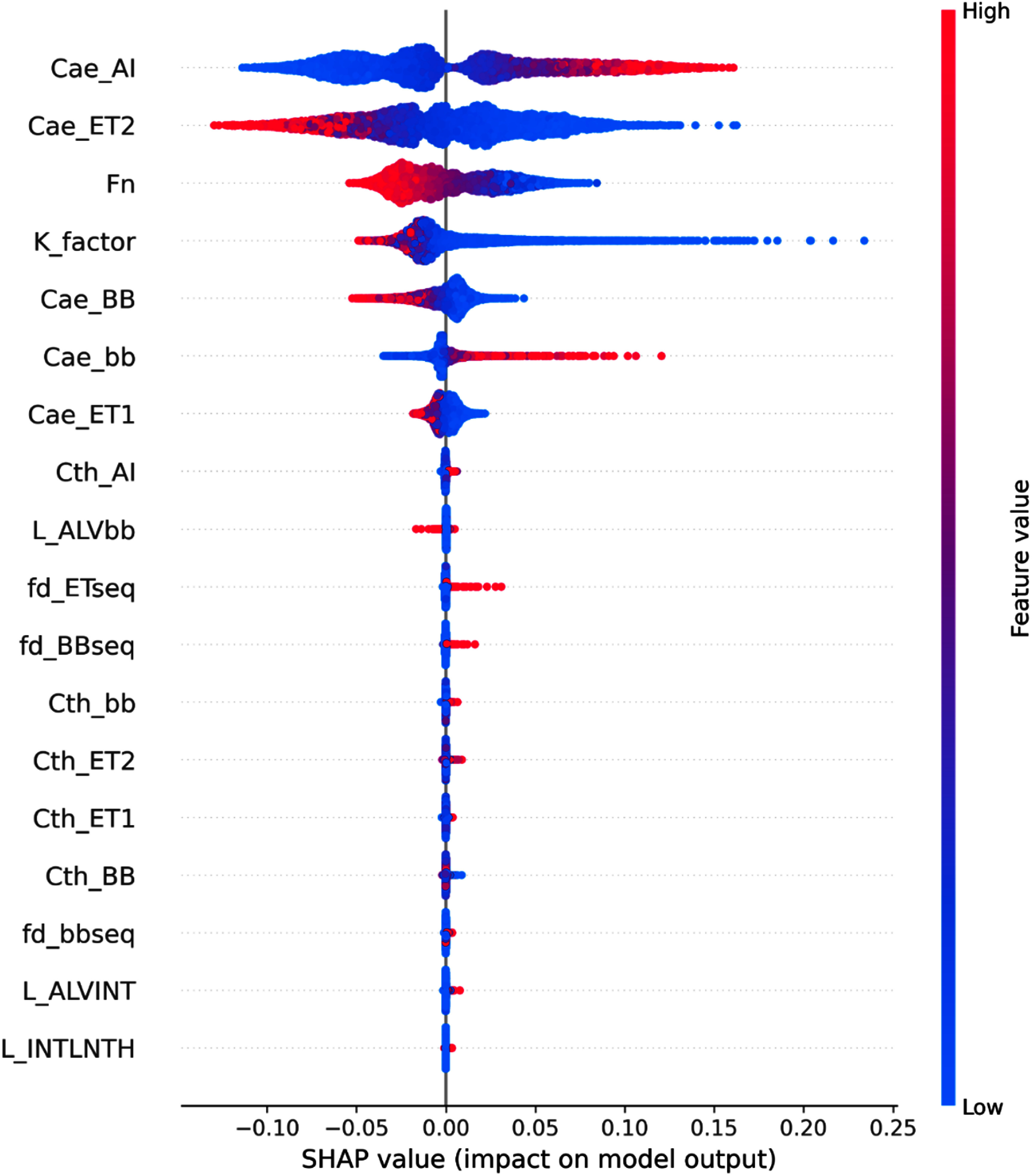
Random forest, integrated with SHAP, parameter importance ranking of uncertain parameters with the committed effective dose coefficient as the target response for extended explanation for ^238^Pu type M.

The machine-learning analysis for the ^238^Pu type M inhalation case was performed using a RF regression model, with a grid search of 200 optimal estimators. Figure 6 presents the resulting ranking of most important parameters for this scenario. The *x*-axis lists the uncertain input parameters, while the *y*-axis represents their corresponding feature importance as determined by the RF model. Figure 7 provides a complementary and comprehensive SHAP-based interpretation of the machine learning model, illustrating the contribution of each parameter to variations in the CEDC. Model performance metrics indicated strong predictive capability. For the training dataset, the MAE and RMSE were 0.0063 and 0.0099, respectively. For the test dataset, the corresponding MAE and RMSE were 0.0175 and 0.0296. Because these values correspond to the normalised scale, the errors were also converted back to the original units of the dose coefficient for interpretation. On the original scale of the CEDC, the MAE was $1.30 \times {10^{ - 6}}Sv/Bq{\text{ }}$ for the training dataset and $3.60 \times {10^{ - 6}}Sv/Bq$ for the test dataset. The MAE values correspond to approximately 0.63% and 1.75% of the CEDC, respectively, indicating that the prediction error remains small relative to the magnitude and variability of the estimated dose coefficient. Across both the standard RF model and the SHAP-incorporated analysis, the parameter identified as most influential for the ^238^Pu type M case was $Cae\_AI$, representing the random variable used as a multiplier of a fitting parameter (a) applied to the aerodynamic deposition efficiency in the AI region within the ICRP deposition model.

**Figure 8.**
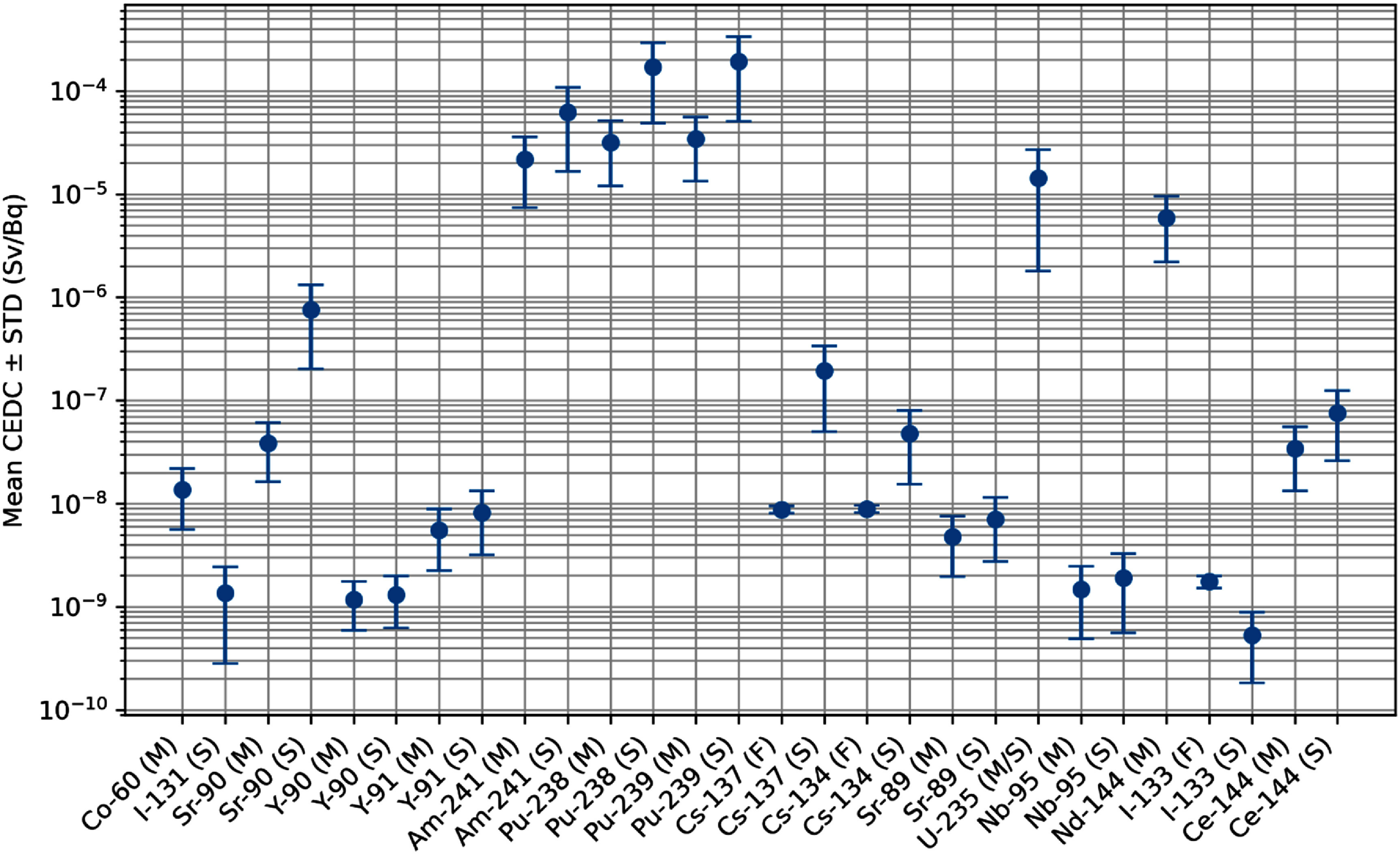
Mean CEDC values with ±1 standard deviation (STD) error bars for the full set of radionuclides and absorption types evaluated. The error bars are calculated in linear space; their apparent asymmetry reflects the use of a logarithmic axis, which was applied to accommodate the wide range of values encompassing different orders of magnitude.

The compendium results of the uncertainty propagation and sensitivity analysis for all the radionuclides and associated absorption types examined in this study are presented in figure 8 and table 5. Figure 8 illustrates the variation of the CEDCs about the mean value (mean ± standard deviation), whereas table 5 summarises the principal statistical findings obtained from the analyses. The RF feature importance rankings were generally stable across radionuclides considered and were consistent with SHAP interpretations. In particular, parameters associated with lung deposition and transport related processes consistently emerged as dominant contributors to dose variability. The transport related processes included the transport scaling parameter (*K_factor*), which was used to jointly scale selected transport rates within the respiratory tract model.The machine learning results complement the physical interpretation and further confirm the known sensitivity of distal lung deposition.

**Table 5.** Statistical results derived from the uncertainty and sensitivity analysis for the complete set of radionuclides evaluated.

Radionuclide	Type	Sample Size	Mean (*Sv*/*Bq*)	STD (*Sv*/*Bq*)	UF	Best fit distribution	Top Three (3) Impactful Parameters
^60^Co	M	10 000	1.37 × 10^−08^	8.11 × 10^−09^	5.36	Gamma	Cae_AI, Cae_ET2, Fn
^131^I	S	10 000	1.36 × 10^−09^	1.08 × 10^−09^	7.12	Lognormal	K_factor, Cae_ET2, Cae_bb
^90^Sr	M	10 000	3.87 × 10^−08^	2.25 × 10^−08^	5.19	Johnsonsb (bounded)	Cae_AI, Cae_ET2, Fn
^90^Sr	S	10 000	7.57 × 10^−07^	5.56 × 10^−07^	10.84	Weibull_min	Cae_AI, Cae_ET2, L_ALVbb
^90^Y	M	5000	1.18 × 10^−09^	5.87 × 10^−10^	3.81	Johnsonsb (bounded)	Cae_ET2, K_factor, Cae_AI
^90^Y	S	5000	1.30 × 10^−09^	6.75 × 10^−10^	4.01	Johnsonsu (unbounded)	Cae_ET2, K_factor, Cae_AI
^91^Y	M	10 000	5.56 × 10^−09^	3.31 × 10^−09^	5.25	Pearson type 3	Cae_AI, Cae_ET2, Fn
^91^Y	S	10 000	8.22 × 10^−09^	5.02 × 10^−09^	5.51	Gauss hypergeometric(gausshyper)	Cae_AI, Cae_ET2, Fn
^241^Am	M	10 000	2.17 × 10^−05^	1.43 × 10^−05^	7.16	Generalised hyperbolic (genhyperbolic)	Cae_ET2, Cae_AI, K_factor
^241^Am	S	10 000	6.20 × 10^−05^	4.55 × 10^−05^	10.17	Pearson type 3	Cae_AI, Cae_ET2, Fn
^238^Pu	M	10 000	3.16 × 10^−05^	1.96 × 10^−05^	6.58	Gamma	Cae_AI, Cae_ET2, Fn
^238^Pu	S	10 000	1.70 × 10^−04^	1.21 × 10^−04^	27.05	Lognormal	Cae_ET2, K_factor, Cae_AI
^239^Pu	M	10 000	3.45 × 10^−05^	2.12 × 10^−05^	6.12	Gamma	Cae_AI, Cae_ET2, Fn
^239^Pu	S	10 000	1.92 × 10^−04^	1.41 × 10^−04^	30.78	Non-central fisher (ncf)	Cae_ET2, K_factor, Cae_AI
^137^Cs	F	10 000	8.73 × 10^−09^	6.98 × 10^−10^	1.31	Generalised hyperbolic (genhyperbolic)	Cae_AI, Cae_ET2, Cae_BB
^137^Cs	S	10 000	1.93 × 10^−07^	1.43 × 10^−07^	10.75	Gauss hypergeometric (gausshyper)	Cae_AI, Cae_ET2, L_ALVbb
^134^Cs	F	10 000	8.90 × 10^−09^	7.16 × 10^−10^	1.31	Skew-normal (skewnorm)	Cae_AI, Cae_ET2, Cae_BB
^134^Cs	S	10 000	4.79 × 10^−08^	3.24 × 10^−08^	8.44	Gauss hypergeometric (gausshyper)	Cae_AI, Cae_ET2, Fn
^89^Sr	M	10 000	4.77 × 10^−09^	2.80 × 10^−09^	5.32	Pearson type 3	Cae_AI, Cae_ET2, K_factor
^89^Sr	S	10 000	7.07 × 10^−09^	4.32 × 10^−09^	5.51	Gamma	Cae_AI, Cae_ET2, Fn
^235^U	M/S	5000	1.43 × 10^−05^	1.25 × 10^−05^	8.43	Lognormal	Cae_AI, K_factor, Cae_ET2
^95^Nb	M	10 000	1.48 × 10^−09^	9.92 × 10^−10^	5.91	Johnsonsu (unbounded)	K_factor, Cae_ET2, Cae_bb
^95^Nb	S	10 000	1.91 × 10^−09^	1.35 × 10^−09^	6.39	Johnsonsu (unbounded)	K_factor, Cae_ET2, Cae_AI
^144^Nd	M	10 000	5.86 × 10^−06^	3.65 × 10^−06^	6.13	Gauss hypergeometric (gausshyper)	Cae_AI, Cae_ET2, Fn
^133^I	F	10 000	1.75 × 10^−09^	2.27 × 10^−10^	1.47	Johnsonsb (bounded)	K_factor, Cae_AI, Cae_BB
^133^I	S	10 000	5.33 × 10^−10^	3.50 × 10^−10^	5.28	Johnsonsu (unbounded)	K_factor, Cae_ET2, Cae_BB
^144^Ce	M	10 000	3.43 × 10^−08^	2.10 × 10^−08^	6.07	Gamma	Cae_AI, Cae_ET2, Fn
^144^Ce	S	10 000	7.57 × 10^−08^	4.96 × 10^−08^	7.26	Johnsonsb (bounded)	Cae_AI, Cae_ET2, Fn

STD: standard deviation; UF: Uncertainty factor.

Overall, several consistent cross cutting trends emerged across the radionuclides and clearance absorption types evaluated in this study. Radionuclides with slow absorption characteristics generally exhibited broader predicted dose coefficient distributions and higher UFs relative to the corresponding fast and moderate absorption types. Across both the statistical analyses and machine learning interpretations, respiratory tract deposition and transport related parameters consistently emerged as the dominant contributors to variability in the inhalation dose coefficients. Among these, parameters associated with the AI and ET2 regions, as well as the fraction of air inhaled (Fn) and K_factor, demonstrated particularly strong sensitivity.

## 4. Discussion

The primary objective of this work was to examine the variability inherent in conventional deterministic particle deposition and biokinetic models with associated dosimetric framework, and to precisely characterise the stochastic behaviour of radionuclides following realistic nuclear security-related intake scenarios. To address limitations associated with fixed parameter frameworks, this study extended traditional methodologies by incorporating stochastic representations of key model inputs across a range of radiological and nuclear source terms. The results section presented a detailed record of analysis for ^238^Pu type M inhalation scenario, followed by a comprehensive summary of findings for the full set of radionuclides evaluated. The uncertainty bounds adopted in this study were selected to reflect realistic population level variability rather than individual physiological extremes and were sampled independently using LHS approach.

The UFs reported in this study represent HRTM-driven uncertainty only, with systemic biokinetic transfer coefficients, gastrointestinal absorption fractions, and dosimetric S coefficients fixed at ICRP reference values. Consequently, the reported uncertainty propagation results should be interpreted as reflecting HRTM-driven uncertainty only and not as representing total internal dosimetry uncertainty. Inclusion of additional uncertainty sources from systemic biokinetics, gastrointestinal absorption, and dosimetric modelling would be expected to likely increase the overall variability of predicted dose coefficients. However, these additions would not fundamentally alter the dominant influence of respiratory deposition and clearance parameters, particularly in inhalation intake scenarios where initial deposition patterns strongly govern subsequent dose outcomes. Additionally, neglecting correlation of the regional deposition uncertain parameters, which is consistent with prior uncertainty studies, may modestly affect the variance but represents a reasonable first order assumption.

The uncertainty and sensitivity analysis of the CEDC for the inhaled ^238^Pu type M reveals noticeable variability across the sampled uncertain parameter space. The deterministic ICRP reference dose coefficient for this scenario ($1.2 \times {10^{ - 05}}{\text{ }}Sv/Bq$) was found to lie below the 25th percentile of the simulated distribution, yet above the 2.5th percentile. This placement indicates that the reference value occupies the lower portion of the predicted uncertainty range based on the uncertain parameters. The corresponding UF (UF = 6.58) as presented in table 4 further suggests that the upper bound of probable dose coefficient outcomes exceeds the ICRP reference point estimate by approximately a factor of seven. In general, the statistical results suggest that the deterministic ICRP dose coefficient underestimates both the median and mean values derived from the probabilistic analysis for inhaled ^238^Pu type M. Although the reference value remains within the 95% confidence interval, the right-skewed distribution and magnitude of the UF feature the potential for substantially higher dose estimates when realistic variability in model parameters is considered.

To characterise the form of the response distribution, a nonparametric K–S test was employed. For the inhaled ^238^Pu type M case, the gamma distribution provided the best representation of the simulated CEDC data (figure 3), yielding a minimum difference of *D*_max_ (${D_{{\mathrm{max}}}}$ = 0.007) between empirical and theoretical cumulative distributions and an associated *p*-value of 0.626. Under the two-sided hypothesis framework, the *p*-value exceeds the 5% significance threshold, indicating no statistical basis to reject the null hypothesis that the empirical data follow the gamma distribution. Comparison of cumulative distribution functions (figure 4) confirmed excellent agreement between the theoretical and simulated curves. The Q–Q plot (figure 5), which was employed to interpret the quality of the fits for the probability distribution functions, likewise supported the adequacy of the gamma fit relative to other candidate distributions, which consistently showed larger deviations.

The sensitivity analysis provided further knowledge of the drivers of the variability. The application of a RF regression model (figure 6), interpreted through SHAP analysis (figure 7), identified the aerodynamic deposition multiplier in the AI region (*Cae_AI*) of the deposition model as the dominant contributor to variance in the inhalation CEDC for the ^238^Pu type M scenario. Both the RF feature importance ranking and SHAP-based ranking yielded consistent results. Variations in *Cae_AI*, which act as a scaling factor on the aerodynamic deposition fitting parameter in the ICRP respiratory tract model, exerted the strongest influence on predicted dose outcomes. This finding is consistent with the modelling complexity of the distal regions of the respiratory tract, where deposition and retention processes strongly influence long-term dose. The SHAP analysis further demonstrated that lower values of *Cae_AI* tend to reduce the predicted CEDC, whereas higher values increase it, with a more pronounced upward influence. Because this parameter directly modifies the deposition efficiency formulation derived from regression analyses of experimental data and theoretical modelling, variability in *Cae_AI* propagates substantially through the dose calculation. Consequently, uncertainty associated with aerodynamic deposition in the AI region represents a principal source of stochastic variation for inhaled ^238^Pu type M.

These findings for ^238^Pu type M provide a representative illustration of how parameter variability influences inhalation dose coefficients. The following discussion extends this interpretation to the full set of the radionuclides and absorption types considered (figure 8 and table 5), highlighting similarities and contrasts in uncertainty magnitude and dominant sensitivity contributors across source terms. The expanded results across the full set of radionuclides and absorption types reveal several consistent patterns in both the magnitude and form of the uncertainty in the inhalation CEDCs. The mean values of the CEDC span approximately five orders of magnitude (difference between the lowest and largest orders of magnitude), reflecting the combined influence of radionuclide decay characteristics, biokinetic behaviour, and regional deposition dynamics. Superimposed on this wide range of central estimates is substantial variability, with UFs ranging from approximately 1.3–30, depending on radionuclide and absorption type. The observed differences between the mean and median values of the CEDC distributions in this study and the reference ICRP CEDC point estimates are likely attributable to the following factors. Firstly, the uncertain parameters used in this study were specifically related to the human respiratory tract. The sensitivity analysis was conducted by simultaneously considering all the uncertain parameters in table 2, while keeping all systemic and dissolution rates constant at their ICRP reference values. Additionally, uncertainties in the dosimetric (*S*-coefficient) model were not included in this study. Furthermore, the distribution assigned to the fraction of air inhaled through the nose (*Fn*) was based on measurements of inter-subject variability among nose-breathing adults. In this study, *Fn* was modelled using a triangular distribution with a minimum value of 0.4 and a maximum value of 1.0. This assumed distribution could partially explain the slight offset of the ICRP reference dose coefficient compared to the mean of the simulated results, since the ICRP point estimate is derived under the assumption of predominantly nasal breathing with *Fn* = 1.0. These findings demonstrate the importance of explicitly accounting for uncertainty and inter-individual variability in dose assessment models to improve applicability under realistic exposure conditions.

A clear trend emerges when comparing fast (F), moderate (M), and slow (S) absorption types. For many radionuclides, particularly actinides and selected fission products, the type S material consistently exhibits larger standard deviations and noticeably higher UF than the corresponding type F and M. This pattern is especially pronounced for high dose actinides, where extended retention in the respiratory tract amplifies the influence of deposition and clearance uncertainties. Importantly, these elevated UFs primarily reflect compounded uncertainty propagation associated with lung deposition, long residence times, and slow clearance kinetics characteristic of slowly soluble inhaled materials.

In contrast, several type F cases (e.g., radioiodine and caesium isotopes) show comparatively narrow distributions and low UF values, indicating that rapid systemic transfer reduces the sensitivity of the CEDC. For example, the deterministic ICRP dose coefficient for ^137^Cs type F case ($9.3 \times {10^{ - 09}}{\text{ }}Sv/Bq$) lies near the upper quartile of the predicted distribution, positioned between the 50th and 75th percentiles (table 5). This placement indicates that the reference dose coefficient of ^137^Cs type F is well aligned with the central tendency of the distribution, with only modest differences attributable to parameter variability in the respiratory tract model. The UF obtained (UF = 1.31) is the smallest among the radionuclides studied, reflecting the relatively low sensitivity of ^137^Cs type F dose predictions to uncertainties in respiratory tract parameters. In contrast, the UF obtained for ^239^Pu type S is the largest among all radionuclides evaluated, indicating that the upper bound dose estimate exceeds the corresponding reference ICRP dose coefficient by approximately a factor of 30.

The probability distribution characterisation analysis further confirms that the inhalation dose coefficients deviate from normality. Across the radionuclides considered, best-fit distributions include gamma, lognormal, Johnson families (bounded and unbounded), skewnorm, Pearson type 3, Weibull, and generalised hyperbolic forms. Under the two-sided hypothesis framework of the K–S test, the p-values exceeded the 5% significance threshold, indicating no statistical basis to reject the null hypothesis that the empirical data follow the respective best-fit distributions. Accordingly, Q–Q plots were used to interpret and validate the quality of the fits. The frequent identification of skewed and heavy-tailed distributions confirms that variability in model parameters propagates asymmetrically into the dose metric. In several type S cases, the pronounced right-skewness of the simulated distributions contributes substantially to the magnitude of the UF, indicating that certain combinations of deposition and clearance parameters can yield dose estimates that extend above the central tendency. This observation has practical implications for consequence management applications, where upper-bound dose projections frequently inform protective action and operational decision-making. The sensitivity analysis results show a dominant role of deposition-related parameters in most inhalation scenarios. In particular, the aerodynamic deposition multiplier in the AI region (*Cae_AI*) was identified as the most influential parameter for the majority of radionuclides. This consistent ranking emphasises the importance of distal lung deposition in determining long-term retention and dose. Moreover, if narrower parameter bounds were applied, the magnitude of the resulting UFs would be expected to decrease without changing the qualitative trends and dominant influence of respiratory tract deposition and clearance related parameters.

Overall, the compendium analysis confirms that uncertainties in regional deposition, especially within the AI and ET2 regions, and in the fraction of air inhaled (Fn) remain the principal contributors to variability in the inhalation dose coefficients across a broad spectrum of nuclear source terms based on the uncertain parameters considered.

## 5. Conclusions

Inhalation exposures resulting from nuclear or radiological security incidents present substantial challenges for internal dose assessment. Estimation of dose coefficients requires integration of respiratory tract deposition, systemic biokinetics, and dosimetric models within a unified framework. Although internationally adopted reference models provide consistency in calculations, they represent idealised individuals and do not explicitly reflect inter-individual variability and general uncertainty in physiological and realistic exposure-related parameters. The objective of this work was to quantify the variability embedded within deterministic biokinetic and dosimetric models and to extend these models to incorporate stochastic parameter representations for nuclear security-relevant radionuclide source terms. As a result, an expanded set of probabilistic inhalation dose coefficients was derived.

The study was conducted in three stages. First, the HRTM described in ICRP Publication 130, together with radionuclide-specific systemic models, was reconstructed in a Python-based computational environment for a compendium of radionuclides. This implementation enabled calculation of particle deposition, retention, excretion, organ doses, and CEDCs for the selected radionuclide source terms. Secondly, model parameters subject to uncertainty were identified and characterised using probability distributions consistent with available experimental and modelling evidence. In the third phase, these uncertainties were propagated through the coupled inhalation biokinetic-dosimetric framework using LHS as the Monte-Carlo sampling engine. The resulting CEDC distributions were examined statistically, and sensitivity was quantified using RF regression coupled with SHAP for comprehensive machine learning interpretation to identify dominant contributors to the model variability. The results demonstrated that parameter uncertainty can significantly influence inhalation dose coefficients, particularly for the radionuclides with slow absorption characteristics. Although deterministic reference dose coefficient values generally fall within the simulated 95% intervals, the magnitude of the UFs indicates significant variability, spanning approximately 1.3–30 depending on radionuclide and absorption type. Across the radionuclides evaluated, the deposition parameter associated with the AI region frequently emerged as the primary contributor to the variability in the inhalation dose coefficients, consistent with the modelling complexity of the lower generations of the respiratory tract.

The stochastic framework developed in this study supports the application of probabilistic dose coefficients and dose quantities to complement conventional deterministic estimates for scenarios that require realistic characterisation of variability. The resulting percentile distributions and UFs are therefore most appropriate within the context of scenario analysis, consequence management, emergency response planning, and retrospective dose reconstruction, where uncertainty informed assessment provides additional insight beyond deterministic reference coefficients in contrast to routine regulatory compliance. In this context, probabilistic coefficients may help identify the range and percentile of potential dose outcomes, establish confidence intervals around reference estimates, and evaluate the sensitivity of inhalation dose predictions to uncertain parameters beyond what is represented by single deterministic dose coefficients.

## Data Availability

All data that support the findings of this study are included within the article.
